# Right lung transplantation with a left-to-right inverted anastomosis in a rat model

**DOI:** 10.1016/j.xjon.2022.01.020

**Published:** 2022-02-09

**Authors:** Heng Huang, Hao-Ji Yan, Xiang-Yun Zheng, Jun-Jie Wang, Hong-Tao Tang, Cai-Han Li, Dong Tian

**Affiliations:** a Heart and Lung Transplant Research Laboratory, Affiliated Hospital of North Sichuan Medical College, Nanchong, China; b Academician (Expert) Workstation, Affiliated Hospital of North Sichuan Medical College, Nanchong, China

**Keywords:** cuff technique, inverted anastomosis, rat, right lung transplantation, situs inversus, AR, acute rejection, CR, chronic rejection, IRI, ischemia–reperfusion injury, LDLLTx, living-donor lobar lung transplantation, LTx, lung transplantation, PA, pulmonary artery, PV, pulmonary vein

## Abstract

**Objective:**

Right lung transplantation in rats has been attempted occasionally, but the technical complexity makes it challenging to apply routinely. Additionally, basic research on inverted lobar lung transplantation is scarce because of the lack of a cost-effective experimental model. We first reported right lung transplantation in a rat model using left-to-right inverted anastomosis to imitate the principle of clinically inverted lung transplantation.

**Methods:**

Right lung transplantation was performed in 10 consecutive rats. By using a 3-cuff technique, the left lung of the donor rat was implanted into the right thoracic cavity of the recipient rat. The rat lung graft was rotated 180° along the vertical axis to achieve anatomic matching of right hilar structures. Another 10 consecutive rats had received orthotopic left lung transplantation as a control.

**Results:**

All lung transplantation procedures were technically successful without intraoperative failure. One rat (10%) died of full pulmonary atelectasis after right lung transplantation, whereas all rats survived after left lung transplantation. No significant difference was observed in heart-lung block retrieval (8.6 ± 0.8 vs 8.4 ± 0.9 minutes), cuff preparation (8.3 ± 0.9 vs 8.7 ± 0.9 minutes), or total procedure time (58.2 ± 2.6 vs 56.6 ± 2.1 minutes) between the right lung transplantation and standard left lung transplantation groups (*P* > .05), although the cold ischemia time (14.2 ± 0.9 vs 25.5 ± 1.7 minutes) and warm ischemia time (19.8 ± 1.5 vs 13.7 ± 1.8 minutes) were different (*P* < .001).

**Conclusions:**

Right lung transplantation with a left-to-right inverted anastomosis in a rat model is technically easy to master, expeditious, and reproducible. It can potentially imitate the principle of clinically inverted lung transplantation and become an alternative to standard left lung transplantation.

## Graphical abstract

**Figure:**
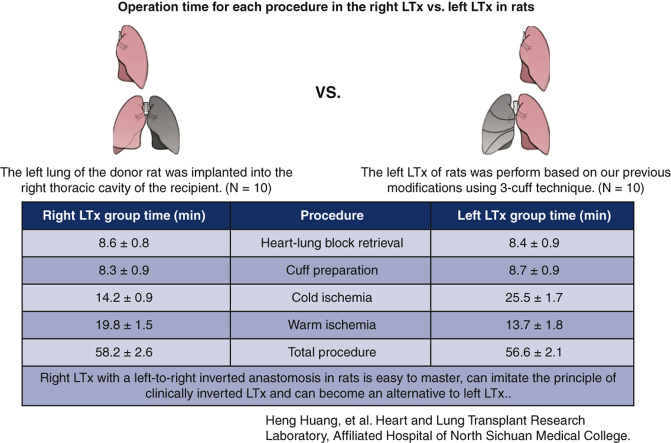

**Figure:**
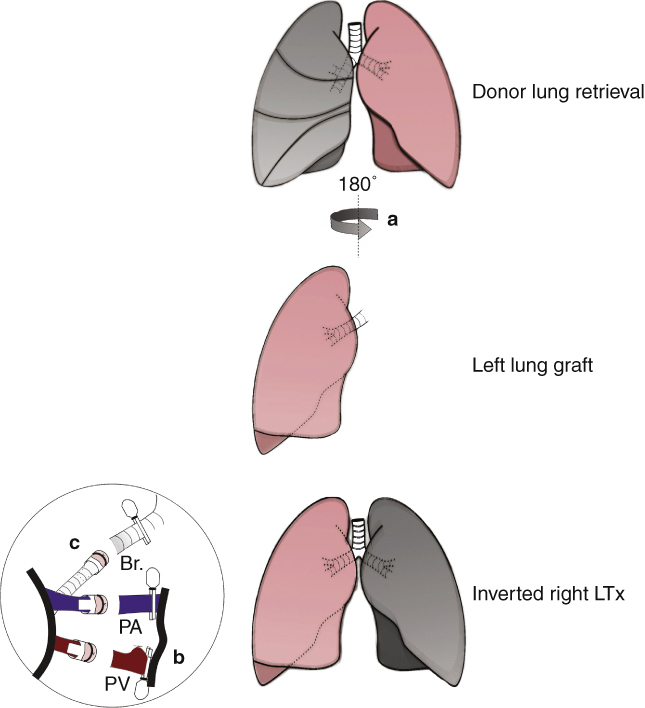
Right LTx with a left-to-right inverted anastomosis in a rat model.

**Central Message**

Right LTx with a left-to-right inverted anastomosis in rats is easy to master, can imitate the principle of clinically inverted LTx, and can become an alternative to left LTx.

**Perspective**

Right LTx with a left-to-right inverted anastomosis in rats is technically easy to master and reproducible, and may serve as an alternative to left LTx for anastomotic expeditiousness and a high success rate. In particular, in inverted techniques, this model can help evaluate pathophysiological mechanisms and translate promising therapeutic research into clinically inverted LTx.

See Commentary on page 440.

Animal models have been the cornerstone of translating preclinical research into life-prolonging strategies throughout the history of lung transplantation (LTx).^1^, ^2^, ^3^ Therein, orthotopic left LTx in rodents (mainly rats and mice) has become the most common procedure since the inception of the cuff technique by Mizuta and colleagues.^4^^,^^5^ Notably, recipient animals after left LTx can survive on the native right lung and manifest as healthy even after allograft necrosis.^6^^,^^7^ Occasional studies have attempted orthotopic right LTx with right allografts in rodents, followed by left pneumonectomy, to alleviate excessive compensation from the native lung.^6^^,^^8^ However, the complex anastomosis and high intraoperative mortality made this right LTx model challenging to apply routinely. Additionally, the standard orthotopic left LTx in small rats will become more difficult for novice surgeons, whereas right LTx may be a good choice because of the larger hilar structures, indicating the necessity of an alternative model of standard left LTx in rats.

Kyoto University pioneered the concept of right-to-left inverted lobar LTx to circumvent the obstacle of size mismatching and donor shortage in living-donor lobar LTx (LDLLTx).^9^^,^^10^ The core of this procedure involved implanting an inverted right lower lobe into the left thoracic cavity, inspiring the current right LTx model.^11^^,^^12^ Compared with traditional LTx, the mechanisms of postoperative pathophysiological variations in inverted LTx are not well understood and include ischemia–reperfusion injury (IRI), acute rejection (AR), and chronic rejection (CR). Experimental animal models have laid a solid and necessary foundation for the further study of left-to-right LTx. Although left-to-right LTx in an expensive large animal model (eg, canine) was used by Okayama University,^13^ the cost, ethical issues, and rigorous management might frustrate scientists considerably. Small rodent animals with a lower price and syngeneic backgrounds can investigate the mechanisms of disease onset and progress reproducibly in inverted LTx.

We report an easy-to-master and reproducible right LTx model with situs inversus in rats, integrating the surgical aspects of the cuff technique and concept of the left-to-right inverted anastomosis technique (Figure 1). Using this right LTx model, we aimed to establish an experimental model to imitate the principle of clinical practice to investigate pathophysiological variations after inverted LTx or LDLLTx and provide an alternative to standard rat left LTx for routine basic research.

**Figure 1:**
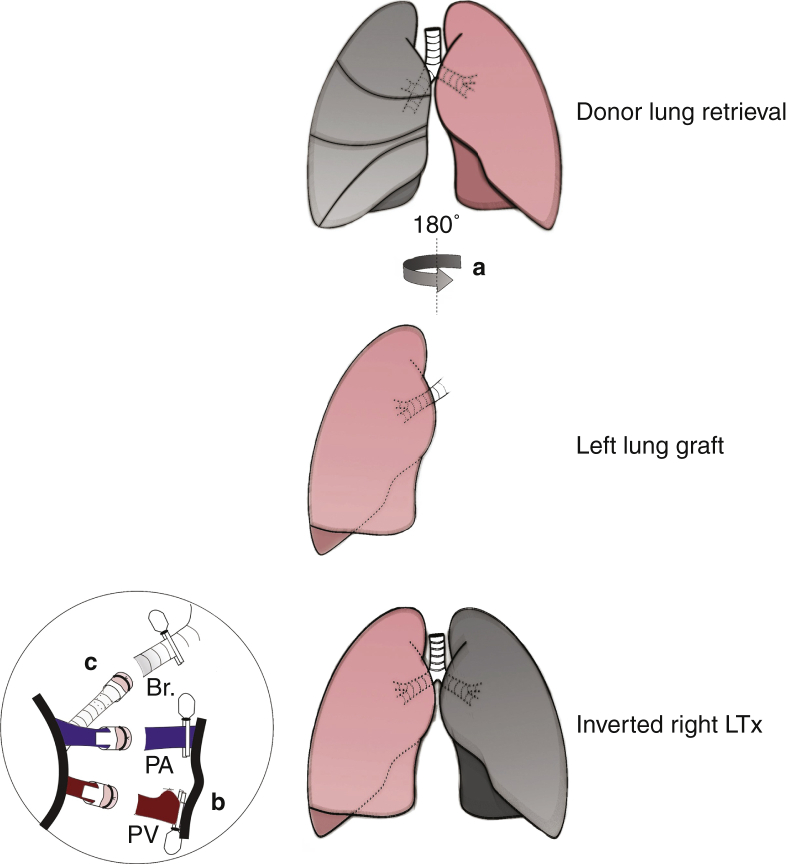
Right LTx with a left-to-right inverted anastomosis in a rat model. A, The left lung of the donor rat was retrieved, rotated 180° along the vertical axis, and then implanted into the right thoracic cavity. B, The superior branch of the PV was ligated and transected. The venous cuff was inserted into the inferior branch of the PV and anastomosed. C, The tail of the bronchial cuff was oriented toward the anterior margin, opposite to that of PA and PV. *Br.*, Bronchus; *PA*, pulmonary artery; *PV*, pulmonary vein; *LTx*, lung transplantation.

## Materials and Methods

### Animals

Lewis rats aged 8 to 10 weeks served as donors, and those aged 10 to 12 weeks served as recipients (Beijing Vital River Laboratory Animal Technology Co, Ltd). All the rats were fed a standard diet with water ad libitum and fasted 1 day before surgery. The operation conditions were clean rather than nonaseptic. This study was approved under License Number 2021-No. 09 from the Experimental Animal Ethics Committee of North Sichuan Medical College. All the procedures followed the Institutional Animal Care and Use Committee of North Sichuan Medical College guidelines.

### Reagents and Devices

Most of the reagents, small handheld microsurgical instruments, and larger equipment for left LTx in rats have been described by us previously.^14^ The material supplier for the current study differs from that used previously. Some important homemade devices were introduced in our technique. Briefly, the tracheal intubation device was assembled from 2 wooden boards and a cambered metal plate, which facilitates exposure of the glottis and the success rate of intubation. The cuff-preparation plate comprised 2 foam blocks with 1 groove and 1 hole and a Petri dish, allowing cuff preparation to be more stable and expeditious without unnecessary lung injury due to compression. All the cuffs were made of 14-G venous indwelling needles (a diameter of 2 mm for the pulmonary artery and vein and 2.1 mm for the bronchus) (Table E1).

### Operation of Standard Orthotopic Left Lung Transplantation

Standard orthotopic left LTx in rats served as a control. The orthotopic left LTx in rats was consistent with that in our previous study, which modified the traditional left LTx model in diverse aspects.^14^

### Recipient Operation of Right Lung Transplantation: Dissection

The recipient rat was anesthetized, orally intubated with a homemade tracheal intubation device, and ventilated with an anesthesia concentration of 5 parts per million (ppm). It had a respiration rate of 90 times/min and a tidal volume of 2.5 to 3 mL. After right thoracotomy, at the apex impulse of the heart (0’17” in Video 1), we dilated the incision using a translational eyelid opener and pulled the 4 lobes (anterior, middle, posterior, and accessory lobes) of the right lung outward using sterile cotton swabs. If the hilum was poorly exposed, another thoracotomy or transection of a lower rib was performed. The anesthetic concentration was decreased to 3 ppm.

**Figure 2:**
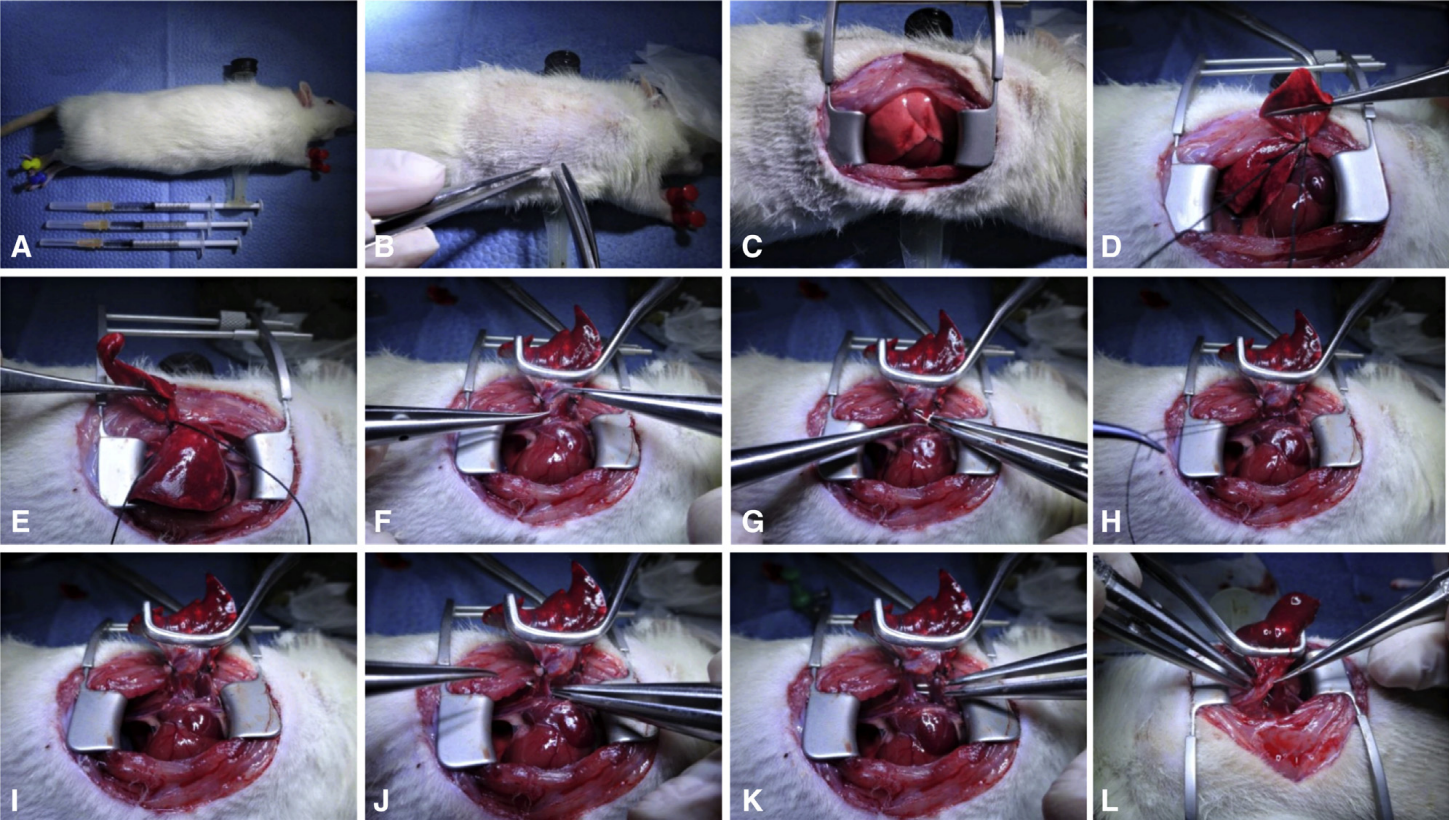
Recipient operation of right LTx: dissection. A, Position of donor rat. B, Point of incision. C, Exposure of thoracic cavity. D, Ligation and discard of the anterior lobe. E, Ligation and discard of the accessory lobe. F, Clamping of hilar structures with nondestructive sidewall forceps and dissection of the inferior PV. G, Dissection of the superior PV. H, Ligation and transection of the superior branch of the PV. I, Structures in the front view plane. J, Dissection of the overlap between the PV and bronchus. K, Dissection of the overlap between the PA and bronchus. L, Dissection of the bronchial and proximal extra tissue in the reverse view plane.

We ligated and discarded the anterior and accessory lobes (0’30” in Video 1). Nondestructive sidewall forceps were used to clamp the right hilar structures. The inferior and superior branches of the vein and artery were observed from left to right. The bronchus was behind vessels. The tidal volume of the ventilator was turned down appropriately to avoid contralateral pulmonary hyperventilation. In the front view plane, we dissected the hilar structures and enlarged the space between the superior/inferior pulmonary artery (PA) and pulmonary vein (PV). We ligated and transected the superior branch of the PV (1’30” in Video 1). We dissected the overlap between the PV, PA, and bronchus using a Stevens nerve hook (1’57” in Video 1). Next, the bronchus and proximal extra tissue were dissected in the reverse view plane. The recipient rat was placed aside temporarily using a ventilator until the right graft was prepared (Video 1 and Figure 2).

### Donor Operation of Right Lung Transplantation

**Figure 3:**
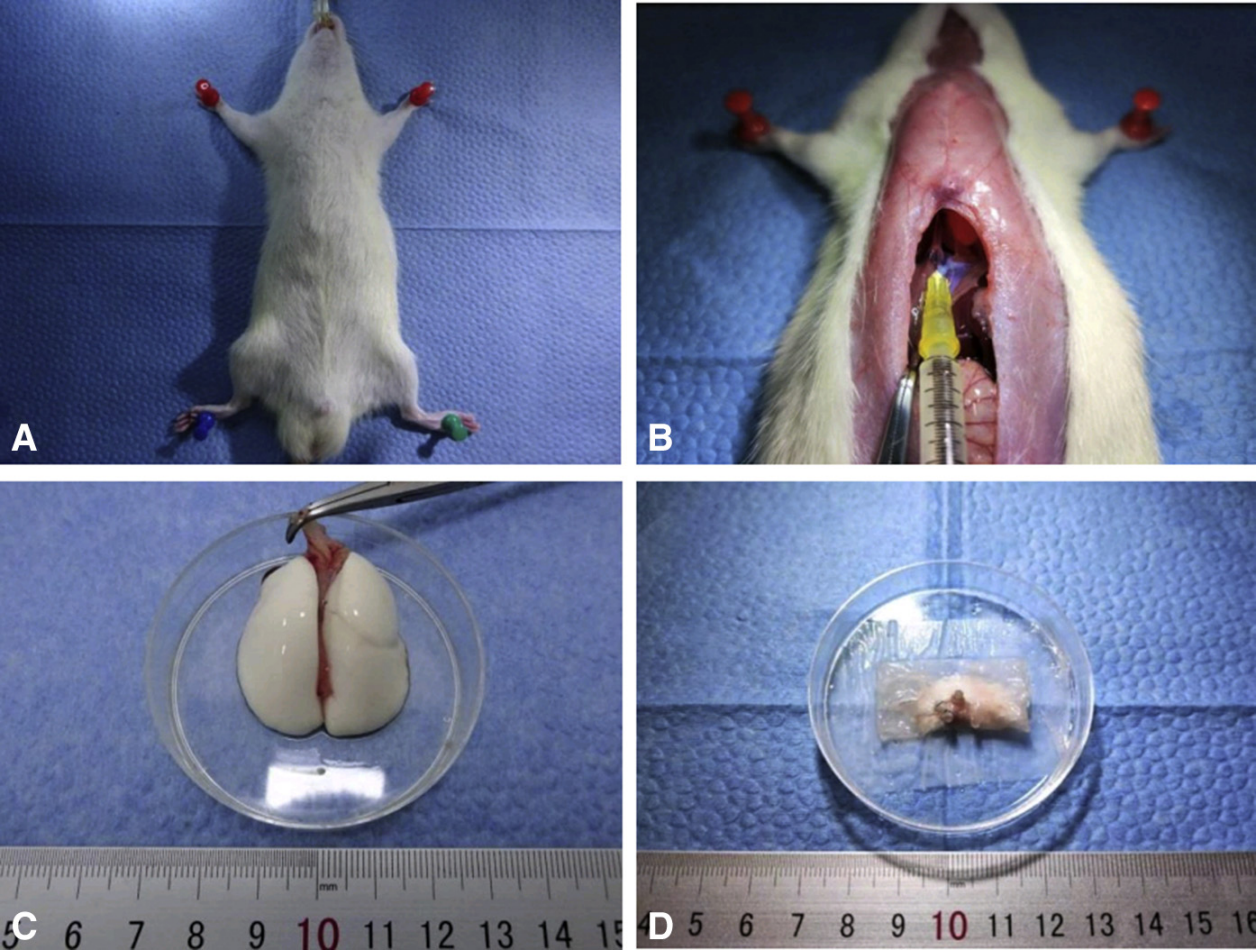
Donor operation of right LTx. A, Position of donor rat. B, Injection of heparin sodium into the inferior vena cava. C, Heart-lung block of donor rat. D, Donor lung block with absorbent paper soaked in ice-cold ET-Kyoto solution. The tails of the PV and PA cuffs must be oriented toward the posterior margin of the lung.

All steps of the donor operation were performed as reported in our previous study.^14^ We offer some critical points to consider: The volume of the left donor lung should be slightly smaller than that of the right thoracic cavity because a moderate donor-recipient size mismatch makes anastomosis easier. Furthermore, the bronchus should be transected with a segment set aside as long as possible, while the PV should be transected along with partial atrial tissue to guarantee a sufficient length for subsequent cuff preparation and anastomosis. Additionally, the tails of the PA and PV cuffs must be oriented toward the posterior margin of the lung, whereas the tail of the bronchial cuff is oriented toward the anterior margin (Figure 3).

### Recipient Operation of Right Lung Transplantation: Anastomosis

**Figure 4:**
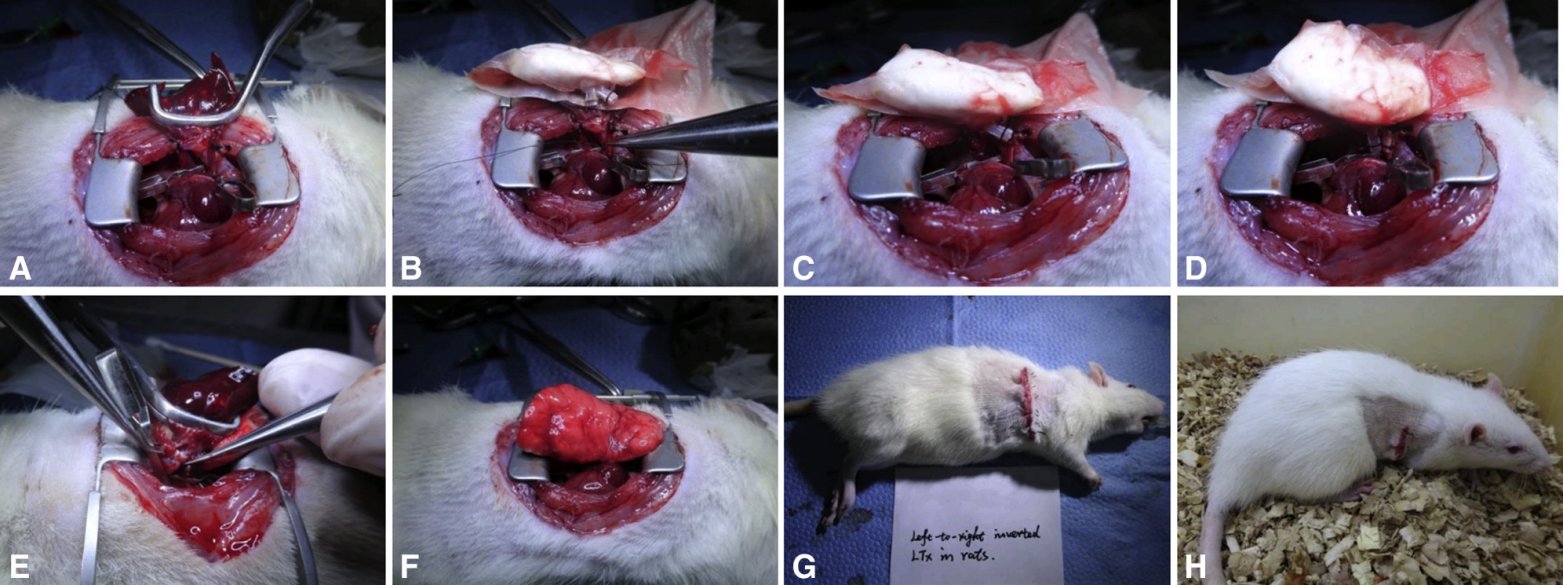
Recipient operation of right LTx: anastomosis. A, Clamping of the PV and PA proximally and draining of blood from the lumen. B, Placement of circumferential 8-0 Prolene around the PV. C, Cuff insertion and venous anastomosis of the right lung. D, Placement of circumferential 8-0 Prolene around the PA and cuff insertion and arterial anastomosis. E, Placement of circumferential 8-0 Prolene around the bronchus and cuff insertion and bronchial anastomosis. F, Restoration of ventilation and perfusion of the right donor lung. G, Suturing of the muscle and skin layers of the right chest. H, Completion of the operation and anesthesia recovery.

We clamped the PV and PA proximally using 2 microvascular clamps (2’42” in Video 1). Transverse incisions were performed on the anterior wall of vessels distally to drain blood from the lumen. The allograft was removed from the low-temperature environment, where the anterior margin of the donor was oriented dorsally while the apex pulmonis was oriented to the cephalad during anastomosis (2’56” in Video 1). A circumferential 8-0 Prolene suture was placed around the PV and loosened. After pulling the anterior wall of the venous incision and clamping the cuff tail with forceps, the venous cuff was inserted into the recipient vascular lumen as close as possible to the proximal part (3’23” in Video 1). After that, we secured the previously placed circumferential Prolene with 2 knots. We transected the distal part of the residual PV and then anastomosed the PA as described (4’05” in Video 1). The hilar structures were then switched to the reverse-view plane. After blocking ventilation with a microvascular clamp and excluding secretions in the bronchial lumen (5’12” in Video 1), the bronchus was anastomosed as described. If the lumen of the bronchus was large, a microvascular clamp was placed to clamp the cuff tail together with the bronchus to guarantee that the cuff would not slip out before securing. The microvascular clamp was never released before reperfusion. We released the microvascular clamp to restore ventilation and achieved reperfusion for 3 to 5 minutes (6’03” in Video 1). We closed the chest and sutured the muscle and skin layers. The rat was weaned off the anesthesia, and partial tracheal intubation was pulled out (Video 1 and Figure 4).

### Postoperative Care

Furosemide, dexamethasone, atropine, and cefazolin were injected intraperitoneally. After transplantation, the recipient rats received a standard diet and regular detection of abnormal behaviors such as convulsions and mobility. Postoperative care generally did not include additional complex therapeutic strategies.

### Postoperative Assessment

The average weight, food intake, and mental state of the rats were recorded daily. An autopsy examination was conducted to investigate possible causes of death. All the recipient rats were killed 1 week after transplantation. After the rats were killed, lung graft tissue of the right LTx and standard left LTx groups were used for routine histological assessment.

### Statistical Analysis

All the results were analyzed using SPSS Statistics, version 26.0 (IBM). The data were described as frequencies, percentages, and means ± standard deviation. Student *t* test was applied to analyze the differences in a certain variable between the right LTx and standard left LTx groups.

## Results

### Outcomes of Right Lung Transplantation

In the current study, the weights of the donor and recipient rats were 260.9 ± 3.2 g versus 310.3 ± 5.5 g in the right LTx group and 231.57 ± 5.94 g versus 254.22 ± 4.77 g in the standard left LTx group at the time of transplantation, respectively. The activity, food intake, and mental state of the recipient rats were approximately the same in the 2 groups. All the procedures of right and left LTx achieved technical success (10 consecutive rats). No intraoperative failure occurred, such as vessel laceration, twisting, cuff slipping, air leakage, anastomotic bleeding, or death. The restoration of ventilation and reperfusion were successful. Neither congestion nor thrombosis was observed in the process.

**Figure 5:**
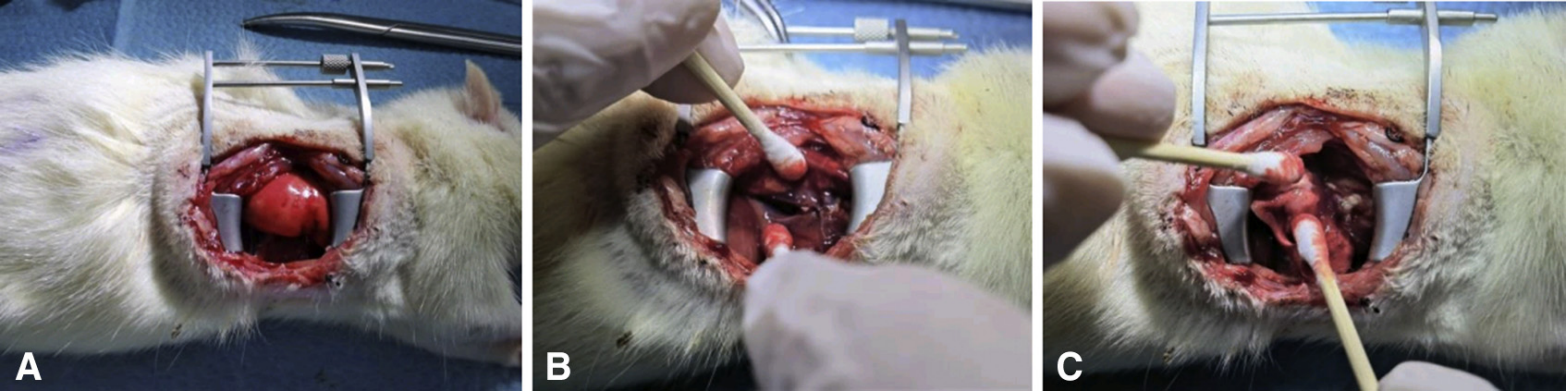
Gross appearance of right LTx 1 week postoperatively. A, No pulmonary atelectasis, pleural effusion, or hemothorax was observed in the thoracic cavity. B, No congestion, twisting, or bleeding was found on the anastomoses of the PA and PV. C, No congestion, twisting, or air leakage was found on the anastomosis of the bronchus.

**Figure 6:**
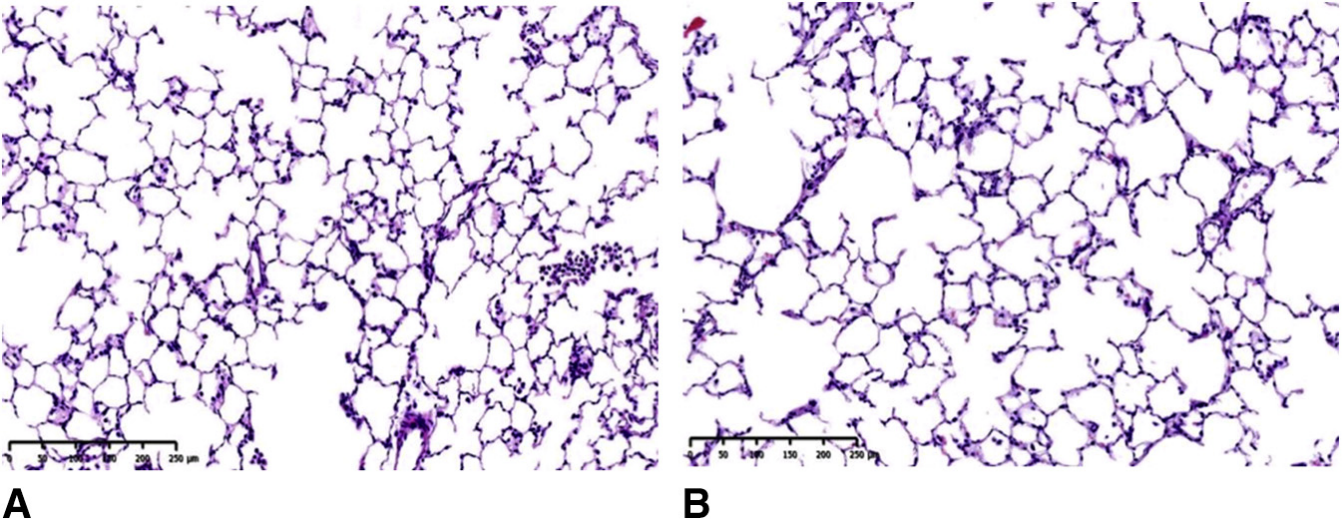
Histological assessment after sacrifice. A, Lung graft tissue from the right LTx group. B, Lung graft tissue from the standard left LTx group. Regular pulmonary parenchyma, structure, and complete alveoli. No rejection was observed in any of the rats after syngeneic transplantation (hematoxylin–eosin; ×100).

On the second postoperative day, 1 recipient rat (10%) in the right LTx group died of full pulmonary atelectasis on the second postoperative day, whereas all rats in the standard left LTx group survived. No pleural effusion, hemothorax, severe infection, or pneumothorax was found in our series after they were killed (Figure 5). No abnormal variation was observed in the structures of the alveoli, vessels, or small airways of the right LTx group or standard left LTx group according to the histological assessment, such as extended inflammation, acute lung injury, rejection, infection, and other pulmonary complications (Figure 6).

### Operation Time of Right Lung Transplantation

**Figure 7:**
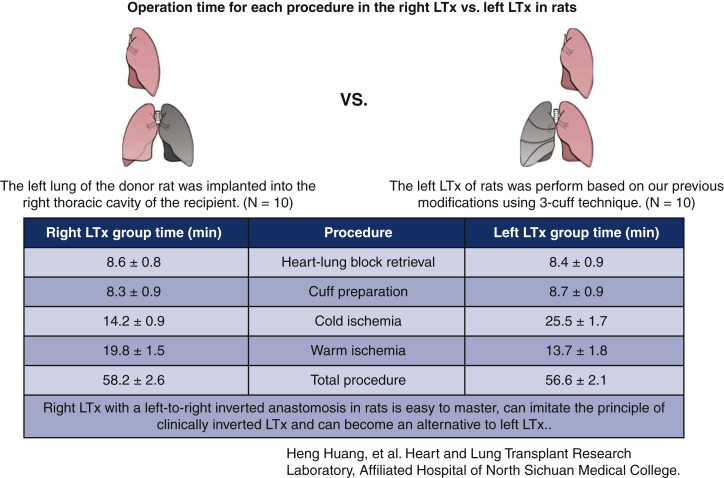
Graphical abstract. No significant difference was observed in heart-lung block retrieval, cuff preparation, or the total procedure time between the right LTx and standard left LTx groups (*P* > .05), although the cold ischemia time and warm ischemia time were different (*P* < .001). *LTx*, Lung transplantation.

No significant difference was found in heart-lung block retrieval (8.6 ± 0.8 vs 8.4 ± 0.9 minutes), cuff preparation (8.3 ± 0.9 vs 8.7 ± 0.9 minutes), or total procedure time (58.2 ± 2.6 vs 56.6 ± 2.1 minutes) between the right LTx and standard left LTx groups (*P* > .05). The cold ischemia time of the right LTx group (14.2 ± 0.9 minutes) was significantly shorter than that of the standard left LTx group (25.5 ± 1.7 minutes) (*P* < .001). By contrast, the warm ischemia time of the standard left LTx group (13.7 ± 1.8 minutes) was significantly shorter than that of the right LTx group (19.8 ± 1.5 minutes) (*P* < .001). The definition of the time interval and some details concerning the operation time are presented in Table 1 and Figure 7.

**Table 1:** Operation time for each procedure

Procedure	Operation time (min)		*P* value
	Right LTx group	Standard left LTx group	
Heart-lung block retrieval∗	8.6 ± 0.8	8.4 ± 0.9	.63
Cuff preparation†	8.3 ± 0.9	8.7 ± 0.9	.36
Cold ischemia‡	14.2 ± 0.9	25.5 ± 1.7	<.001
Warm ischemia§	19.8 ± 1.5	13.7 ± 1.8	<.001
Total procedureǁ	58.2 ± 2.6	56.6 ± 2.1	.15

*LTx*, Lung transplantation.

∗ Heart-lung block retrieval: from midline laparotomy to excision of the donor heart-lung block.

† Cuff preparation: from excision of the heart-lung block to completion of cuff placement.

‡ Cold ischemia: from flushing the donor lungs to graft removal from hypothermic storage.

§ Warm ischemia: from donor lung removal from ice to reperfusion.

ǁ Total procedure time: from the donor's skin incision to closure of the recipient's incision.

## Discussion

A previous orthotopic rat right LTx model with a right allograft has been established, but it has not been developed further because of technical challenges.^6^^,^^7^ Because of the necessity of right LTx and considering the inspiration from right-to-left inverted LDLLTx, we first reported on the right LTx model using a left-to-right inverted anastomosis in a rat model. This model was easy to master and technically expeditious; using this inverted anastomosis technique, one author (J.-J.W.) completed the first successful right LTx at the eighth attempt, which was comparable to the result of our previous modifications in left LTx in rats.^14^ Considering the reproducible procedures with a short learning curve, the current model can serve as an alternative model of standard left LTx.

Kawaguchi and colleagues^8^ first introduced a right LTx model in rats in its anatomic position. However, it was challenging to anastomose all hilar structures quickly because of the anatomic complexity, resulting in a long learning curve. Li and colleagues^6^ established a similar right LTx model in mice, in which 2 venous branches of the donor lung were combined and then anastomosed to 1 branch of the native PV. However, the right main PV was too short to be everted over the cuff, likely causing the cuff to slip off during anastomosis. In summary, the operational challenges in the 2 right LTx models revealed the difficulties in the routine use of these procedures in genetic and mechanistic studies.

Our current model is the first to introduce left-to-right inverted LTx in rats, in which the inverted left donor lung is implanted into the right thoracic cavity. Primarily, the left lung of the rat contains only 1 lobe; thus, the procedure is easy to perform, as we previously reported.^14^ Additionally, a moderate mismatch in donor-recipient patients makes anastomosis technically easier. The donor lung selected in the current model is generally smaller than that of the right thoracic cavity, making it conducive to anastomosis and donor-recipient size matching.^15^^,^^16^ Additionally, previous literature has reported the rudiments of inverted LTx, documenting the physiological feasibility of anastomosis in its nonanatomic position in both animals and humans and providing an opportunity to develop a small animal platform to further study clinically inverted LTx.^9^^,^^10^^,^^17^^,^^18^ Notably, we previously described the key points and modifications of diverse aspects of the procedure to optimize the rat orthotopic left LTx model.^14^ The time for cuff preparation and warm ischemia (∼8 minutes for each) was shortened by half compared with other reports. Based on the existence of the left-to-right inverted technique, the advantage in time will be extended to the current right LTx model.

In previous studies, acute hemorrhage caused by cuff slipping and vascular injury was the main cause of intraoperative death in rats, leading to an overall survival of less than 90%.^19^^,^^20^ Three recipients (7.5%) died of intraoperative bleeding in a previous study on right LTx by Li and colleagues^6^ likely because of the short length of the main right PV that was everted over the cuff. By contrast, no intraoperative failure or death occurred in the current model. Thus, our procedure might directly benefit from the easy anastomosis of large and thick hilar structures. During the follow-up period, the overall survival of recipient rats in this study was 90%, slightly higher than that in Li and colleagues' study (87.5%). One recipient rat (10%) died of full pulmonary atelectasis. Atelectasis is one of the most severe complications after LTx in rats and directly leads to graft necrosis and failure.^14^ According to our autopsy, this death may have been related to bronchial angulation after closing the chest. A bronchial cuff with a larger diameter and ligation closer to the tracheal carina can be the practical solution. Overall, the survival in this study was satisfactory based on the inherent difficulty of dissection and anastomosis in the right LTx.

The operation time is one of the determinants of whether one LTx model can be applied on a large scale. Overall, the difference between the operation time of standard left LTx in the current study (56.6 ± 2.1 minutes) and our previous study (48.0 ± 2.8 minutes) was acceptable for individual proficiency.^14^ Notably, no significant difference was found in the total operation time between left LTx and right LTx in this study, and the times were significantly superior to those of other previous modified models (>80 minutes).^19^^,^^21^ Specifically, heart-lung block retrieval and the cuff preparation time (∼8 minutes) in the right LTx group were comparable to those in the standard left LTx group, mainly because no difference was found between the 2 transplantation models except in cuff size during the preparation of the donor lung. By contrast, in the studies of Guo and colleagues^19^ and Habertheuer and colleagues,^20^ the cuff preparation times were 18.7 minutes and 20 minutes, respectively. Ischemia of allografts will trigger an imbalance between the metabolic supply and demand, and eventually contribute to tissue hypoxia, cell damage, or death.^22^ Therefore, the ischemic time has been an essential factor influencing the risk of IRI and graft dysfunction. Kawaguchi and colleagues^8^ anastomosed the superior PV, inferior PV, PA, and bronchus in the right LTx, aggravating the anastomotic complexity and resulting in an ischemia time (cold plus warm ischemia time) of up to 69 ± 8 minutes. However, our model simplified the anastomosis procedure and reduced the ischemia time to 33 minutes (14.2 ± 0.9 for cold ischemia and 19.8 ± 1.5 for warm ischemia), a decrease of over half. Because of the inherent challenge of right lung anatomy, the warm ischemia time during anastomosis was slightly longer than that of left LTx in our previous study. However, the single left lobe and large right hilar lumen provide technical convenience for anastomosis in our model, making the warm ischemia time shorter than the previously reported time (∼15-20 minutes).^19^^,^^20^^,^^23^ Given the time difference caused by the anastomosis technique and sequence, comparing cold and warm ischemia times in controlled settings between right and left LTx is futile. Thus, we recommend comparing the total ischemia time when considering the procedure time.

Concerning scientific use, this model is easier to master for novice surgeons with a high success rate because cuff insertion and anastomosis are easier. According to the current study results, inverted right LTx has similar perioperative results and operation times to the standard left LTx we previously reported. Therefore, this model may serve as an alternative for standard left LTx for short-term observation and may be applied in genetic and mechanistic studies of IRI, AR, and CR and in some therapeutic regimens, particularly in the cases of novice surgeons and small rats.^2^^,^^24^, ^25^, ^26^, ^27^ Additionally, although the inverted anastomosis technique was a breakthrough in clinical LTx in Japan, whether the mechanisms of postoperative pathophysiological variations are the same as those of traditional LTx remains uncertain.^11^^,^^12^ To our best knowledge, generalized inverted LTx has only been performed in canine models before clinical success and had the drawbacks of a high cost and complicated genetic background.^13^ The current small rodent model that is inexpensive and genetically better defined can be established to assess the pathophysiological mechanisms and allow more rapid accrual of experimental data in genetic studies before large-scale clinical application.^5^ Although right LTx in rats is indeed noncongruent from human LTx in lung and chest anatomy, it can serve as an indicator for genetic and mechanistic studies of inverted LTx or LDLLTx. Furthermore, orthotopic left LTx in mice plays an essential role in genetic studies because of abundant transgenic and knockout strains, but the hilar structures are too small to be anastomosed well.^5^ The thicker right hilar structures provide an opportunity for inverted LTx models in mice to become routine experimental tools in genetic therapy. We will attempt to apply the technique of inverted anastomosis to right LTx in mice in the future. Finally, small animals undergoing bilateral LTx survive on the graft alone to better imitate clinical bilateral sequential LTx, which is currently the most frequently applied technique worldwide. However, bilateral LTx in rodents has failed to achieve success temporarily because of technical challenges. Li and colleagues^6^ attempted to perform bilateral sequential LTx in mice, resulting in the death of animals during the operation; they believed that success was impossible using current microsurgical techniques. The current method of implanting the left lung into the right thoracic cavity of rats substantially simplifies the surgical procedure, promoting the process of bilateral LTx in rodents.

### Study Limitations

Several limitations of the current study should be mentioned. First, the current study emphasized the description of surgical techniques and potential scientific use; no information was provided on assessing graft function or postoperative disease models, including AR, CR, and IRI. Additionally, the donor lung selected in this study was slightly smaller than the thoracic cavity of the recipient rats to achieve size matching and expeditious anastomosis. However, this matching in small animal LTx was largely based on macroscopic estimation, making it difficult to conduct quantitative studies on rat donor-recipient size matching. Finally, the potential of assessing immunologic studies was not definite because of the short-term follow-up time (7 days). However, according to the previous experience of the modified left LTx model,^14^ most deaths occurred within 1 week postoperatively. The recipient rats that have survived 1 week may continue to survive for mid- and long-term studies.

## Conclusions

Right LTx with a left-to-right inverted anastomosis in a rat model is technically easy to master and reproducible, potentially making it an alternative choice to the standard left LTx because of anastomotic expeditiousness, a high success rate, and the physiological nature. In particular, in the field of inverted anastomotic techniques, this model can help evaluate pathophysiological mechanisms and translate promising therapeutic research into clinically inverted LTx or lobar LTx.

### Ethical Statement

The authors are accountable for all aspects of the work in ensuring that questions related to the accuracy or integrity of any part of the work are appropriately investigated and resolved. Experiments were performed under a project license (No.: 2021-No. 09) granted by the Experimental Animal Ethics Committee of North Sichuan Medical College in compliance with the Institutional Animal Care and Use Committee of North Sichuan Medical College guidelines.

### Conflict of Interest Statement

The authors reported no conflicts of interest.

The *Journal* policy requires editors and reviewers to disclose conflicts of interest and to decline handling or reviewing manuscripts for which they may have a conflict of interest. The editors and reviewers of this article have no conflicts of interest.

## Figures and Tables

**Table E1:** Reagents and instruments

Name	Specification	Company
Inhaled isoflurane	100 mL/bottle	RWD, China
Flushing and cold storage solution for solid organ preservation	1 L	Celsior, France
Heparin sodium	2 mL: 12,500 units	Sinopharm Rongsheng Pharmaceutical Co, Ltd, China
Furosemide	2 mL: 20 mg	Henan Runhong Pharmaceutical Co, Ltd, China
Dexamethasone	1 mL: 5 mg	Sinopharm Rongsheng Pharmaceutical Company, China
Atropine	1 mL: 0.5 g	Tianjin Jinyao Pharmaceutical Co, Ltd, China
Cefazolin	1.0 g	Shenzhen Lijian Pharmaceutical Co, Ltd, China
Nondestructive sidewall forceps	14 cm double angle bend 45° × 14/1 × 2 teeth	Shanghai Jinzhong, China
BARRAQUER spring needle forceps	No teeth/no buckles-bent/13.5 cm	RWD, China
CASTROVIEJO spring scissors	Curved/pointed and pointed/14.5 cm	RWD, China
Microvascular bulldog clamp	Str/3.5 cm	Shanghai Jinzhong, China
Vascular expansion forceps	Angled	Fine Science Tools, British Columbia, Canada
Vessel clamp gripper-with SS micro clamps	Str	RWD, China
SS micro clamps-str	L∗W 6∗1.0 mm/19 mm	RWD, China
Eyelash-shading translation type eyelid opener	14 mm	Shanghai Admiralty, China
VANNAS spring scissors	(Triangular)-S/S angled (6.3∗1.35 mm/8.5 cm)	RWD, China
Stevens nerve hook	6 mm/12.5 cm	RWD, China
Binocular/trinocular stereo microscope	—	RWD, China
VentStar small animal ventilator	—	RWD, China
Flexible veterinary anesthesia machine-wall mount	—	RWD, China
Tracheal intubation device		The device is assembled using 2 wooden boards (25 × 15 × 3 cm) and a metal plate (39 × 15 cm). The surface of the metal plate is curved, and the upward side of the curved plate is equipped with 2 nails of equal height. A 1-0 Prolene with adequate length is connected between the 2 nails.
The catheter of tracheal intubation		The tracheal intubation and metal core comprise 14G venous indwelling needles and are cut into 3.5 cm and 5 cm sizes, respectively. The cusp of the metal core is rounded to ensure to withdraw freely from the venous indwelling needles. This homemade instrument can be used repeatedly.
Cuff-preparation plate		The plate contains a Petri dish attached to 2 foam blocks. A groove and hole are carved on the foam blocks to match the ends of a bulldog clamp.
Cuff		The cuffs of the donor lung comprise 14G venous indwelling needles (a diameter of 2 mm size for the PA and PV, and 2.1 mm for the bronchus), and the surface is sanded roughly to facilitate tight ligation. The cuff comprises the body part and tail part. The cuff body is available in a 2 mm size. The cuff tail is 1.5 mm, the edge of which is curved to prevent accidental injury of the parenchyma or vessels during or after the operation.

*PA*, Pulmonary artery; *PV*, pulmonary vein. The reagents, microsurgical instruments, and surgical animals listed are the preferred specifications in our laboratory. All similar equipment, reagents, and animal strains are alternative and used appropriately according to preferences and operability.

## Supplementary Data

Video 1 Dissection and anastomosis of hilar structures in right LTx with a left-to-right inverted anastomosis in a rat model. Video available at: https://www.jtcvs.org/article/S2666-2736(22)00037-7/fulltext.
