# Supplementary figures and images for: Right lung transplantation with a left-to-right inverted anastomosis in a rat model
Source: JTCVS Open. 2022 Feb 9;10:429–39. doi: 10.1016/j.xjon.2022.01.020 (PMC9390618; doi:10.1016/j.xjon.2022.01.020)

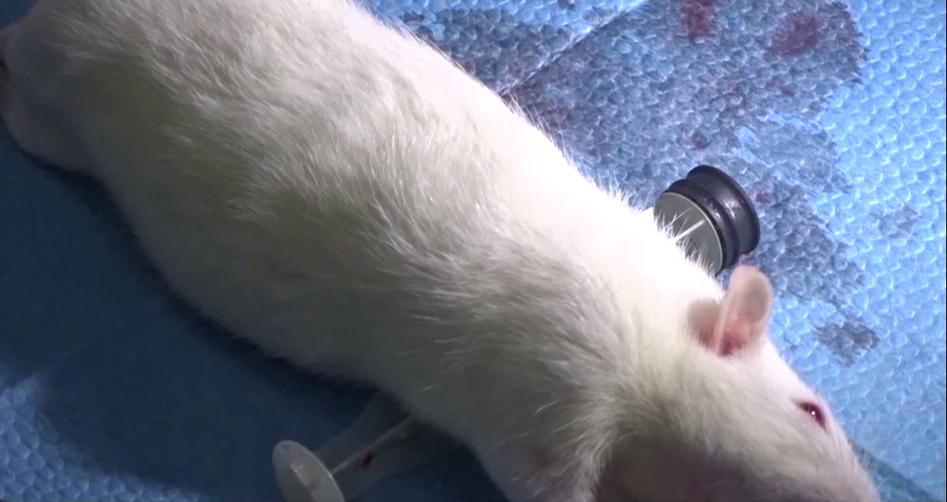

Supplement: Video 1 — Dissection and anastomosis of hilar structures in right LTx with a left-to-right inverted anastomosis in a rat model. Video available at: https://www.jtcvs.org/article/S2666-2736(22)00037-7/fulltext. [file fx3.jpg]
